# Antimicrobial stewardship: knowledge, attitudes and practices regarding antimicrobial use and resistance among non-healthcare students at the University of Zambia

**DOI:** 10.1093/jacamr/dlad116

**Published:** 2023-11-07

**Authors:** Steward Mudenda, Patience Chisha, Billy Chabalenge, Victor Daka, Ruth Lindizyani Mfune, Maisa Kasanga, Martin Kampamba, Phumzile Skosana, Eustus Nsofu, Jimmy Hangoma, Linda Siachalinga, Christabel Nang’andu Hikaambo, Tadious Chimombe, Aurel Constant Allabi, Bawa Boya, Webrod Mufwambi, Zikria Saleem, Scott Kaba Matafwali

**Affiliations:** Department of Pharmacy, School of Health Sciences, University of Zambia, Lusaka, Zambia; Surveillance and Research Technical Working Group, Antimicrobial Resistance, Zambia National Public Health Institute, Lusaka, Zambia; Department of Pharmacy, School of Health Sciences, University of Zambia, Lusaka, Zambia; Department of Medicines Control, Zambia Medicines Regulatory Authority, Lusaka, Zambia; Department of Public Health, Michael Chilufya Sata School of Medicine, Copperbelt University, Ndola, Zambia; Department of Public Health, Michael Chilufya Sata School of Medicine, Copperbelt University, Ndola, Zambia; College of Public Health, Zhengzhou University, 100 Kexue Avenue, Zhengzhou, Henan 450001, China; Department of Pharmacy, School of Health Sciences, University of Zambia, Lusaka, Zambia; Department of Clinical Pharmacy, School of Pharmacy, Sefako Makgatho Health Sciences University, Pretoria, South Africa; Department of Pharmacy, School of Health Sciences, University of Zambia, Lusaka, Zambia; Department of Pharmacy, School of Health Sciences, Levy Mwanawasa Medical University, Lusaka, Zambia; Department of Pharmacy, School of Health Sciences, University of Zambia, Lusaka, Zambia; Department of Pharmacy, School of Health Sciences, Levy Mwanawasa Medical University, Lusaka, Zambia; Department of Pharmacy, School of Health Sciences, University of Zambia, Lusaka, Zambia; Department of Pharmacy, School of Health Sciences, University of Zambia, Lusaka, Zambia; Laboratory of Pharmacology and Toxicology, University of Abomey-Calavi and Teaching Hospital of Abomey-Calavi/Sô-Ava, Abomey-Calavi, Benin; Laboratory of Biology and Molecular Typing in Microbiology, University of Abomey-Calavi, Cotonou, Benin; Department of Pharmacy, School of Health Sciences, University of Zambia, Lusaka, Zambia; Department of Pharmacy Practice, Faculty of Pharmacy, Bahauddin Zakariya University, Multan, Pakistan; Clinical Research Department, Faculty of Infectious and Tropical Diseases, London School of Hygiene & Tropical Medicine, Keppel Street, London WC1E 7HT, UK

## Abstract

**Background:**

Antimicrobial resistance (AMR) poses a significant threat to the world and could become humanity's next major challenge. This study assessed non-healthcare students’ knowledge, attitude and practices (KAP) towards antimicrobial use (AMU) and AMR at the University of Zambia.

**Methods:**

This cross-sectional study was conducted among 443 non-healthcare students from August to October 2022 using a structured questionnaire. Data analysis was done using IBM SPSS version 24.0.

**Results:**

Of the 433 participants, 55.2%, 63.5% and 45% had moderate KAP scores regarding AMU and AMR. The prevalence of self-medication with antibiotics was 76.7%. Male participants were less likely to have good knowledge (OR = 0.524, 95% CI: 0.347–0.792) and positive attitudes (OR = 0.585, 95% CI: 0.364–0.940) towards AMU and AMR compared with females. Students who were studying Engineering and Mining were more likely to have good knowledge of AMR (OR = 1.891, 95% CI: 1.197–2.987) compared with those in Social Sciences. Those who were in their fourth and fifth years were more likely to have positive attitudes towards AMU and AMR (OR = 1.851, 95% CI: 1.147–2.986) compared with those who were in the first, second and third years. Finally, students who practised self-medication were less likely to have good self-reported practice towards AMR (OR = 0.442, 95% CI: 0.278–0.702) compared with those who did not.

**Conclusions:**

This study demonstrated that non-healthcare students had moderate KAP regarding AMU and AMR. All university students should be provided with education about AMU and AMR through free short courses, seminars, workshops, and AMR and antimicrobial stewardship awareness campaigns.

## Introduction

The discovery of antibiotics has saved millions of human lives and increased life expectancy.^1–3^ However, their overuse and misuse has led to the development of antimicrobial resistance (AMR).^4–8^ AMR is a global public health problem that threatens human, animal and environmental health.^9–13^ Antimicrobial-resistant infections cause infections that are difficult or impossible to treat.^5,14^ AMR has far-reaching consequences for both the economy and patient well-being because it can lead to longer hospital stays, higher healthcare expenses and increased risk of death.^5,15–19^ Moreover, AMR has a detrimental effect on global food safety because it jeopardizes the integrity of the food supply chain and can contribute to the spread of antibiotic-resistant bacteria through the food we consume.^20–23^

AMR occurs when disease-causing microorganisms develop the ability to survive even when exposed to antimicrobials that were previously effective at killing or inhibiting their growth.^24,25^ This resistance can arise through gene mutation or gene transfer and has become an increasing public health concern as it can result in treatment failure, leading to increased morbidity and mortality.^24,26,27^ AMR is a complex and multifaceted phenomenon that is influenced by a range of risk factors in both humans and animals.^18,28–30^ These risk factors include self-medication (SM), a lack of diagnostic tools, dispensing of antimicrobials without a prescription, and inappropriate prescribing and administration of antimicrobials.^8,18,28,31–34^ The antimicrobials used in humans belong to the same class as those that are commonly used in animal food production for therapeutic and chemoprophylactic uses.^30,35^ Consequently, the inappropriate use of antimicrobials in agriculture and food animal production can result in the development of resistant microorganisms, which may be passed on to humans through the consumption of meat from the affected animals.^36^ This has led to selection pressure across the One Health ecology.^37,38^

Evidence has shown that knowledge, attitudes and practices (KAP) of individuals regarding antimicrobial use (AMU) and AMR affect the development of antimicrobial-resistant pathogens.^39–41^ This is because individuals who have low KAP tend to use antimicrobials inappropriately thereby increasing the risks of AMR development.^12,40,42^ Alongside this, low awareness of AMU and AMR is also a driver for the emergence of drug-resistant infections.^43^

Addressing AMR requires a collaborative multidisciplinary approach.^25,44–46^ This is because AMR affects all sectors, including humans, animals and the environment.^47^ Additionally, promoting awareness and implementation of antimicrobial stewardship (AMS) programmes are critical in addressing AMR^44,48–52^ as these activities promote the rational prescribing, dispensing and optimal use of antimicrobials^48–50,53^ whilst also increasing awareness of AMU and AMR.^25,54,55^

AMR in Zambia is understudied, and the level of resistance to commonly prescribed antimicrobials is significant across the human, animal and environmental sectors.^11,56–61^ Current evidence from the literature suggests that improved standardization of diagnostic testing of infectious diseases in Zambia could help to better delineate AMR patterns, allowing comparisons across different locations to track AMR evolution over time.^62^ Additionally, coordinated AMS and surveillance programmes have been recommended.^31,63^ This is also built on the Zambian National Action Plan (NAP) on AMR that promotes tackling AMR using a holistic One Health approach and strengthening AMS and surveillance programmes.^64,65^ However, achieving the goals of NAP is difficult because there is a lack of information about the awareness and KAP around AMU and AMR in populations like non-healthcare students and the general public. Therefore, understanding the KAP of university and college students is critical in promoting the rational use of antimicrobials and developing robust strategies to curb AMR.^41,66–68^ Therefore, this study assessed the KAP towards AMU and AMR among non-healthcare students at the University of Zambia. We believe that these findings could help the authorities responsible for implementing strategies that address AMR.

## Materials and methods

### Study design, population and site

This cross-sectional study was conducted at the University of Zambia among non-healthcare students from August to October 2022, according to the STROBE guidelines. The University of Zambia offers non-healthcare training to students in the Schools of Mines, Humanities and Social Sciences, Education, Engineering and Law. For a student to be part of the study, they had to study in a non-healthcare degree programme at the University of Zambia's main campus. Only students who consented to take part in the study were included.

### Sample size estimation and sampling technique

The sample was estimated using Cochran's formula, as previously explained by Charan and Biswas.^69^ With no previous study done among non-healthcare students in Zambia, we employed a conservative prevalence of 50% at a 95% CI and a 5% margin of error. This resulted in a minimum of 385 participants being included in the study. We included a 10% surplus to the sample size to adjust for any incomplete or non-responses, which resulted in a total required sample size of 424 participants. Potential participants were identified through the students’ register. Eligible students were randomly selected using computer-generated random numbers after stratification by year of study.

### Data collection

The data collection tool used in this study was adapted from a previous survey.^70^ The questionnaire had four sections, namely: Section A, which had questions on the students’ socio-demographic characteristics; Section B, which had five questions on the knowledge of students regarding AMU and AMR; Section C, which had five questions on the attitudes of students towards AMU and AMR; and Section D, which had eight questions on the practices of students towards AMU and AMR. Each question had three responses, i.e. ‘Yes’, ‘No’ and ‘I don’t know’. Before commencing the data collection process, the questionnaire was pre-validated for clarity, simplicity, relevance, understandability and accuracy by experts in the field of public health and pharmacy. After face validation, we distributed the questionnaire to 20 non-healthcare students as a pilot study to validate the questionnaire. Some feedback from the pilot study was used to modify and optimize the final structured questionnaire. In the pilot study questionnaire, we used a lot of abbreviations that the non-healthcare students found difficult to remember across different questions. Hence, we removed the abbreviations and wrote all the words in full. With a Cronbach’s α-value of 0.73 that indicated an acceptable internal consistency, the reliability of the questionnaire was assured. Findings from the pilot study were excluded from the analysis of results from the main study. Data collection was physically done by two data collectors (unknown to the students to avoid bias), and it took approximately 20–25 min for a questionnaire to be filled out by each participant.

### Data presentation and analysis

The data collected were entered into Microsoft Excel version 2013 (Microsoft, Redmond, WA, USA) for processing and later exported to IBM Statistical Package for the Social Sciences (SPSS) version 24.0 (IBM, Chicago, IL, USA) for statistical analysis. Descriptive statistics were performed on the socio-demographics, and these results were presented as tables. Five questions were used to assess the knowledge. A ‘Yes’ response was given a score of 3, an ‘I don't know’ response was given a score of 2 and a ‘No’ response was given a score of 1. The total scores for knowledge and attitude were 15 each, whereas those for practice were 24. Scores >80% were regarded as good KAP, scores from 60% to 80% were regarded as moderate KAP, whereas scores ≤60% were regarded as poor KAP. To determine the factors that influenced KAP for students regarding AMR, a univariate analysis was first performed, from which all variables with *P* < 0.2 were used to build the model. Logistic regression was used to determine the factors that influenced the KAP of students regarding AMR. The goodness of fit of the model was assessed using the Hosmer and Lemeshow test (*P* = 0.934). All statistical tests were conducted at a 95% CI, and a *P* < 0.05 was considered statistically significant.

### Ethical approval

The University of Zambia Health Sciences Research Ethics Committee granted ethical approval for this study (protocol ID: 2022112301176). Permission to conduct the research at The University of Zambia was obtained from the University’s management at the respective schools. Strict confidentiality was adhered to throughout the research process; access to the collected data was limited solely to the investigative team. Moreover, to ensure participant anonymity, no personal identifiers were employed in the data collection or analysis stages. Finally, participation was voluntary and therefore non-participating students were not affected academically in any way.

## Results

Of the 433 participants, the majority (51.5%, *n* = 223) were males. Most of the students (31.4%, *n* = 136) were in their fourth year of study and were studying social sciences (72.7%, *n* = 315). SM was reported among 76.7% (*n* = 332) of the students (Table 1).

**Table 1.** Socio-demographic characteristics of participants

Variable	Characteristics	Frequency	Percentage
Sex	Female	210	48.5
	Male	223	51.5
Age (years)	18–25	399	92.1
	>25	34	7.9
Year of study	First	102	23.6
	Second	114	26.3
	Third	73	16.9
	Fourth	136	31.4
	Fifth	8	1.8
Marital status	Married	17	3.9
	Unmarried	416	96.1
Residence	Rural	118	27.3
	Urban	315	72.7
Study programme	Social sciences	315	72.7
	Non-social sciences	118	27.3
A family member works in healthcare	Yes	129	29.8
	No	304	70.2
SM with antibiotics	Yes	332	76.7
	No	101	23.3

Approximately half (50.1%, *n* = 217) of the participants thought that poor use of antibiotics leads to AMR and that bacteria cause the common cold and influenza (53.1%, *n* = 230). Most of the students (53.1%, *n* = 230) knew that if antibiotics are taken frequently, they are less likely to work in the future. Additionally, (46.9%, *n* = 203) agreed that AMR is a global public health problem and compromises the treatment of diseases (Table 2).

**Table 2.** Participants’ knowledge responses regarding AMU and AMR

Participant knowledge	Frequency (*N* = 433)	Percentage
Poor use of antibiotics leads to antimicrobial resistance		
I don’t know	194	44.8
No	22	5.1
Yes	217	50.1
Does antimicrobial resistance mean that if antibiotics are taken frequently, they are less likely to work in the future?		
I don’t know	141	32.6
No	62	14.3
Yes	230	53.1
Do bacteria cause the common cold and influenza?		
I don’t know	96	22.2
No	68	15.7
Yes	269	62.1
Antimicrobial resistance is a global public health problem		
I don’t know	200	46.2
No	30	6.9
Yes	203	46.9
Treatment of diseases is compromised due to the poor use of antibiotics		
I don’t know	132	30.5
No	47	10.9
Yes	254	58.7

The majority of the participants (59.8%, *n* = 259) felt that antibiotics can be used commonly like other medicines. Additionally, 45.5% (*n* = 197) of the participants used antibiotics when they had a cough or sore throat, and 45.5% (*n* = 197) of the students used antibiotics to prevent a cough or sore throat (Table 3). However, 67.7% (*n* = 293) of participating students disagreed that the poor use of antibiotics shortens the duration of illness.

**Table 3.** Participants’ attitude responses regarding AMU and AMR

Participant attitude	Frequency (*N* = 433)	Percentage
Antibiotics are safe drugs, hence they can be commonly used medicines		
I don’t know	78	18.0
No	96	22.2
Yes	259	59.8
Skipping one or two doses of antibiotics does not contribute to the development of antimicrobial resistance		
I don’t know	177	40.9
No	165	38.1
Yes	91	21.0
Adverse effects of antibiotics are reduced by using more than one antibiotic at a time		
I don’t know	188	43.4
No	141	32.6
Yes	104	24.0
Poor use of antibiotics shortens the duration of illness		
I don’t know	95	21.9
No	293	67.7
Yes	45	10.4
Did you take antibiotics as your first-choice drugs to prevent a cough or sore throat?		
I don’t know	97	22.4
No	139	32.1
Yes	197	45.5

This study found that most students (61.7%, *n* = 267) reported that they did not stop taking a course of antibiotics prescribed by the doctor after feeling better. The majority of the participants (56.4%, *n* = 244) kept the remaining antibiotics to use the next time they were sick. Additionally, 76.2% (*n* = 330) of participating students did not discard remaining antibiotics or leftover medicines. Consequently, 49.7% (*n* = 215) of the students gave leftover antibiotics to their friends/roommates to take when they were unwell (Table 4).

**Table 4.** Participants’ practice responses regarding AMU and AMR

Participant practice	Frequency (*N* = 433)	Percentage (%)
Do you stop taking the course of an antibiotic prescribed by a doctor after feeling better?		
I don’t know	19	4.4
No	267	61.7
Yes	147	33.9
Do you keep the remaining antibiotics to use the next time you become sick?		
I don’t know	20	4.6
No	169	39.0
Yes	244	56.4
Do you discard the remaining antibiotics and leftover medicines?		
I don’t know	24	5.5
No	330	76.2
Yes	79	18.2
Do you give leftover antibiotics to your friends/roommates if they get sick?		
I don’t know	14	3.2
No	204	47.1
Yes	215	49.7
Do you complete the full courses of antibiotic treatment?		
I don’t know	15	3.5
No	135	31.2
Yes	283	65.4
Do you consult a doctor or other health workers before starting an antibiotic treatment?		
I don’t know	17	3.9
No	176	40.6
Yes	240	55.4
Do you check the expiry date of the antibiotics before using them?		
I don’t know	17	3.9
No	100	23.1
Yes	316	73.0
Do you prefer to take an antibiotic when you have a cough and sore throat?		
I don’t know	45	10.4
No	130	30.0
Yes	258	59.6

This study found that most of the participants had moderate KAP scores around AMU and AMR (Table 5). However, the students scored better on knowledge questions compared with attitude and practice questions. This entails that their knowledge of AMU and AMR did not translate to similar positive attitudes and good practices.

**Table 5.** Overall knowledge, attitudes and practice regarding AMU and AMR

Variable	Good, *n* (%)	Moderate, *n* (%)	Poor, *n* (%)
Knowledge	181 (41.8)	239 (55.2)	13 (3)
Attitudes	`90 (20.8)	275 (63.5)	68 (15.7)
Practice	132 (30.5)	195 (45)	106 (24.5)

Male students were less likely to have good knowledge (OR = 0.524, 95% CI: 0.347–0.792) and positive attitudes (OR = 0.585, 95% CI: 0.364–0.940) towards AMU and AMR compared with their female counterparts. Students who were studying non-social sciences (Engineering and Mining) were more likely to have good knowledge of AMU and AMR (OR = 1.891, 95% CI: 1.197–2.987) compared with those in social sciences training. Students who were in higher years of studies (fourth and fifth years) were more likely to have positive attitudes towards AMU and AMR (OR = 1.851, 95% CI: 1.147–2.986) compared with those who were in the first, second and third years. Finally, students who practised SM were less likely to have good self-reported practice towards AMU and AMR (OR = 0.442, 95% CI: 0.278–0.702) compared with those who did not practice SM (Table 6).

**Table 6.** Factors influencing knowledge, attitudes and practice of participants around AMU and AMR

Variable	Characteristic	Univariate analysis (*P* value)	Attribute	OR (95% CI)	*P* value
Knowledge	Gender	0.019	Females	1	0.002
			Males	0.524 (0.347–0.792)	
	Programme	0.063	Social sciences	1	0.006
			Non-social sciences	1.891 (1.197–2.987)	
Attitudes	Gender	0.033	Females	1	0.027
			Males	0.585 (0.364–0.940)	
	Year of study	0.017	1–3 years	1	0.012
			4–5 years	1.851 (1.147–2.986)	
Practice	Self-prescription	0.001	No	1	0.001
			Yes	0.442 (0.278–0.702)	

## Discussion

To the best of our knowledge, this was the first study to assess the KAP of non-healthcare students regarding AMU and AMR in Zambia. This study found a high prevalence of SM and moderate KAP concerning AMU and AMR among the study participants.

The prevalence of SM reported in our study (76.7%) is comparable to the 79.3% reported among non-medical students in Saudi Arabia.^71^ This rate of SM was higher than the 68.1% reported in Ethiopia,^72^ 57% in Tanzania,^73^ 54.6% in Nigeria,^74^ 41% in Zambia,^66^ 39.3% in Malaysia^75^ and 37% in Libya.^76^ Our study revealed that one of the causes of a high prevalence of SM could be due to a lack of knowledge concerning AMU and AMR among non-healthcare students, similar to reasons from other studies.^75,77^ However, a higher prevalence of SM has also been reported among medical students, including 98.2% in Saudi Arabia,^78^ 91.7% in Australia,^79^ 89.6% in Iran,^80^ 83% in Pakistan^81^ and 81.3% in Serbia.^82^ The increased practices of SM reported in our study and similar studies could be due to ease of access to antibiotics, as was seen during the COVID-19 pandemic.^68,83^ Therefore, SM remains a problem among university students and requires urgent educational interventions to reduce this issue.^84,85^

Participants in this study demonstrated moderate knowledge overall, a finding that surpasses reported levels in comparable studies among non-medical and non-healthcare students in the United Arab Emirates (UAE)^86^ and Bangladesh,^1^ where knowledge regarding AMU and AMR was generally low. The deficiency of knowledge observed in these cases may reflect a dearth of educational initiatives or awareness programmes concerning AMU and AMR within certain university settings.^41,87^ Conversely, research has indicated a substantial understanding of AMU and AMR among medical and healthcare students in Zambia,^66,67^ Nigeria,^88^ Ghana^89^ and India.^90^ It could be surmised that the apparent disparity in knowledge may be attributed to specific content about AMU and AMR integrated within their healthcare training curricula.^66,67,88,89^

This study further found that most students were aware that AMR is a public health problem. This is consistent with the findings of a study that was conducted among medical students in India, where a significant number of students acknowledged the importance and seriousness of AMR on a global scale.^90^ Interestingly, most participants in our study were aware that the inappropriate use of antibiotics contributes to the development of AMR. These findings are consistent with reports from Colombia, where a high level of awareness was observed among students regarding the link between AMR and the inappropriate use of antibiotics.^91^ Furthermore, most students in our study thought that bacteria were the primary cause of the common cold and influenza. Such misinformation can contribute to the misuse of antibiotics when individuals contract these illnesses. In contrast to this finding, a study in India reported that 79.5% of medical students disagreed with this notion.^90^

Our study found that most students had a moderate score regarding attitudes to AMU and AMR. Our findings contrast with those reported among medical and non-medical students in the UAE, with lower scores on attitude towards AMU and AMR.^86^ Conversely, studies in Zambia and Ecuador found good attitudes towards AMU and AMR,^66,92^ which could be a result of students’ curricula that cover the subject well. The present study also found a lack of awareness concerning skipping doses of antibiotics as a driver of AMR. Similarly, in India many students thought that skipping one or two doses does not lead to the development of AMR.^90^ Our study further found that most students felt that antibiotics were the first choice of treatment for cough and sore throat, but also used them for colds. In line with our findings, a study in the UAE reported that two-thirds of medical students believed that antibiotics can speed up recovery from cough, sore throat, colds and related viral infections.^86^ A study in Jordan indicated that clinicians prescribed antibiotics to treat the common cold, and consequently students were well misguided to think antibiotics could be used in such a manner.^93^ The attitude of non-healthcare students is unsurprising given that the majority of them believed that the common cold was caused by bacteria. This misconception was reported to be the cause of the misuse of antibiotics among students in Nigeria^88^ and Jordan.^93^ In contrast, the majority of medical students in Zambia did not use antibiotics to treat congested nostrils and headaches caused by the common cold.^67^ This calls for more emphasis during awareness campaigns and sensitization programmes on the cause of the common cold and the conditions in which antibiotics are used.

The current study revealed that most students scored moderately for practices towards AMU and AMR. Similar observations were seen in a study among pharmacy students at the University of Zambia.^66^ However, poor practices towards AMR and AMU were reported among medical students in Nigeria^88^ and India.^94^ Poor practices regarding antibiotic use and resistance could be due to a lack of AMS programmes in universities and colleges,^87,88^ and gaps observed in other universities.^95–100^ The present study also found that most participants completed antibiotic courses despite feeling better after consuming two to three doses. These findings are comparable to those that were obtained in Bangladesh, where students of non-biology disciplines completed the doses of antibiotics even when they felt well.^1^ However, it is of concern that 31.2% of students in our study did not complete their courses in antibiotic therapy. Similar observations were reported in Malaysia,^41^ Saudi Arabia^71^ and Libya,^76^ where students stopped taking antibiotics after feeling better. Unfortunately, this practice is among the drivers of AMR. Our study also found that some students did not seek medical advice before taking antibiotics. Studies in Bangladesh and Nepal found that more than 90% of the students sought medical advice before taking antibiotics.^1,101^ However, a study in Nigeria found that most students did not consult a doctor before starting antibiotic treatment.^88^ Unfortunately, most students in our study kept leftover antibiotics and offered the remainder to their friends who had fallen sick or kept them for the next time they got sick. This behaviour was also reported in studies in the West Indies,^102^ Jordan^93^ and India,^90^ where most participants kept leftover antibiotics. Leftover antibiotics are a major driver of SM among students, healthcare workers and the general population.^103,104^ Hence, there is a need for education of students about the consequences of SM.^105^

The observed moderate KAP scores for AMU and AMR in this study require educational interventional activities that may promote the students’ awareness and KAP on the subject matter, especially among students with low KAP. Through workshops, seminars, interactive sessions and the integration of AMS programmes into curricula, students can develop a comprehensive understanding of AMU and AMR, fostering responsible practices and contributing to the global fight against AMR.^106–109^ AMS programmes have been reported to improve the knowledge and behaviours of individuals concerning AMU and AMR.^48,50,110–113^

Our study had some limitations. We selected non-healthcare students from one university in Zambia, hence generalization of the findings should be done with caution. Further, the lack of validation of the adapted tool may affect our findings. Also, the use of a cross-sectional study may not predict the outcomes of an intervention. However, our study provides an insight into the KAP of non-healthcare students regarding AMU and AMR, which might influence policymakers to develop strategies to address this problem in this population. Consequently, our study demonstrated the need to provide educational interventional programmes and activities, especially among social sciences students, those in the earlier years of study and those who practise SM.

### Conclusion

This study found that most students had moderate KAP regarding AMU and AMR. Alongside this, the prevalence of SM was high. The findings of this study underscore the need and importance of addressing the KAP concerning AMU and AMR among non-healthcare students, especially among social sciences students in earlier years of study and those who practise SM. By providing targeted educational interventions, non-healthcare students can be empowered to make informed decisions, adopt responsible behaviours, and contribute to global efforts in combating AMR.
